# The complete chloroplast genome of *Primula bulleyana*, a popular ornamental species

**DOI:** 10.1080/23802359.2019.1678436

**Published:** 2019-10-21

**Authors:** Xiong Chen, Li Zhang, Wenqing Li, Yuan Huang, Zhikun Wu

**Affiliations:** a School of Life Sciences, Yunnan Normal University, Kunming, PR China;; b CAS Key Laboratory for Plant Diversity and Biogeography of East Asia, Kunming Institute of Botany, Chinese Academy of Sciences, Kunming, PR China;; c Department of Pharmacy, Guizhou University of Traditional Chinese Medicine, Guiyang, PR China

**Keywords:** Complete chloroplast genome, *Primula bulleyana*, ornamental species

## Abstract

*Primula bulleyana* is a popular ornamental species, with high commercial values. Here, we reported the first chloroplast genome of *P. bulleyana*. The complete chloroplast genome is 150978 bp, containing a large single-copy (LSC) region of 82873 bp, a small single-copy (SSC) region of 17,741 bp, and a pair of inverted repeats (IRs) regions of 25,182 bp. In total, there are 142 genes, 92 protein-coding genes, 8 rRNA genes, and 38 tRNA genes are annotated in the whole cp genome, including 119 unique genes, 82 unique CDSs, 29 unique tRNAs, and 4 unique rRNAs. The overall GC content of the cp genome is 37.1%. The phylogenetic tree shows that close relationships among *P. bulleyana* and *Primula stenodonta.*

*Primula bulleyana* Forrest is a beautiful species of flowering herbs perennial in the family Primulaceae, native to southwest Sichuan and northwest Yunnan (Lijiang) of China (Hu and Kelso 1996). The species is one of the plants with ornamental value and it has been popular garden plants and crossing parent strain since first introduced by Forrest in 1906. In garden hybrids, *P. bulleyana* is usually as the mother to hybridize with *Primula beesiana,* and the hybrids tending to be yellow or orange (Richards 1993, 2003). In this study, we reported the first chloroplast genome of *P. bulleyana* for understanding its systematics and provide scientific basis for the breeding hybrid of *P. bulleyana* resource in the garden.

The fresh leaves of *P. bulleyana* were collected from Yulong Snow Mountain (Lijiang, Yunnan, China). The voucher specimens (WZK140521) of *P. bulleyana* were deposit at the Herbarium of Yunnan Normal University. Total genomic DNA was extracted using a modified CTAB method (Porebski et al. 1997) and then fragmented and applied to establish short-insert libraries (300 bp), and then the paired-end library was sequenced using Illumina Hiseq X Ten sequencer. The clean data (ca. 36.5 million) were obtained after the removal of low-quality reads and adapter sequences and then were assembled *via* the programme NOVOPlasty version 2.7.2 (https://github.com/ndierckx/NOVOPlasty)(Dierckxsens et al. 2016), with complete chloroplast genome of its close relative *Primula poissonii* as reference (GenBank accession No. KF753634). The assembled chloroplast genome was annotated and adjusted manually using Geneious version 8 (Biomatters Ltd., Auckland, New Zealand) software (Kearse et al. 2012).

The complete chloroplast genome of *P. bulleyana* is 150978 bp in length with an overall GC content of 37.1% (GenBank accession MN428416). The assembled genome containing a large single-copy (LSC) region of 82873 bp, a small single-copy (SSC) region of 17,741 bp, and a pair of inverted repeats (IRs) regions of 25,182 bp. In total, there are 142 genes, 92 protein-coding genes, 8 rRNA genes, and 38 tRNA genes are annotated in the whole cp genome, including 119 unique genes, 82 unique CDSs, 29 unique tRNAs, and 4 unique rRNAs.

Phylogenetic position of *P. bulleyana* was analysed using the maximum likelihood (ML) method. The relative 13 Primulaceae species with complete chloroplast genomes were downloaded from Genbank. The whole chloroplast genome sequence of these species were aligned by the MAFFT (Katoh and Standley 2013); The ML tree was constructed using IQ_TREE 1.6.2 (Nguyen et al. 2015) and performed base on TVM + F+R2 model according to Bayesian information criterion using ModelFinder (Kalyaanamoorthy et al. 2017); ultrafast bootstrap (UFBoot) was used to teste branch supports (Hoang et al. 2018) and SH-like approximate likelihood ratio test (SHAlrt) (Guindon et al. 2010) with 10,000 bootstrap replicates. The phylogenetic tree showed that *P. bulleyana* and *Primula stenodonta* formed a monophyletic clade with 100% bootstrap value and sister to *P. chrysochlora* and *P. poissonii* (Figure 1). The complete chloroplast genome of *P. bulleyana* will provide a genome resource for the breeding of horticultural varieties of Primose as well as for the phylogenetic studies of Primulaceae.

**Figure 1.**
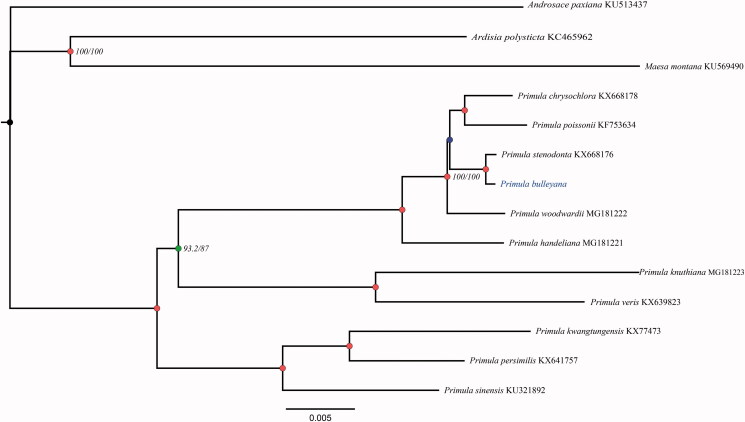
ML phylogenetic tree of *P. bulleyana* and 13 Primulaceae species based on chloroplast complete genome, branch supports values were reported as SH-aLRT/UFBoot, green solid dot denotes supports values of 100/100.
